# Development of Immunoglobulin M Nephropathy in a Pregnant Woman

**DOI:** 10.7759/cureus.20739

**Published:** 2021-12-27

**Authors:** Koray Uludag, Yesim Celik, Nuray Yildirimer, Fatos Tekelioglu, Ali Ihsan Gunal

**Affiliations:** 1 Department of Nephrology, The University of Health Sciences, Kayseri City Training and Research Hospital, Kayseri, TUR; 2 Department of Internal Medicine, The University of Health Sciences, Kayseri City Training and Research Hospital, Kayseri, TUR; 3 Department of Obstetrics and Gynecology, The University of Health Sciences, Kayseri City Training and Research Hospital, Kayseri, TUR; 4 Department of Pathology, The University of Health Sciences, Kayseri City Training and Research Hospital, Kayseri, TUR

**Keywords:** steroid therapy, nephropathy, immunoglobulin m, nephrotic syndrome, pregnancy

## Abstract

Immunoglobulin M nephropathy (IgMN) is a glomerular disease that may be identified in all age groups, but children and young adults appear to have been affected more frequently in some series. The clinical picture could differ from hematuria to rapidly progressive glomerulonephritis. The main characteristics in pathologic examination are mesangial hypercellularity with a diffuse and granular immunoglobulin M deposition in the glomerular structure. To date, a standardized protocol has not been proposed for IgMN treatment. Systemic corticosteroids, calcineurin inhibitors, cyclophosphamide, and rituximab were agents reported in the literature. We present a 30-year-old woman admitted to the hospital for edema in the lower extremities at the 31st week of pregnancy. She had one abortus previously, and this was her second pregnancy. Renal biopsy performed after delivery was reported as IgMN with mesangial proliferation. She received 1 mg/kg/day prednisone therapy achieving complete remission. This report is the first case of IgMN developed in pregnancy.

## Introduction

Immunoglobulin M nephropathy (IgMN) is a rare glomerular disease first introduced in the literature in 1978 [1]. Although the disease can be seen in all ages, it was observed primarily in children and young adults, having a prevalence of 1.8% of native kidney biopsies from the case series reported [2]. The disease appears clinically in varying forms, from hematuria to rapidly progressive glomerulonephritis [3]. There may be mesangial hypercellularity, minimal changes, or sclerosis in glomerular tuft in light microscopy, with a diffuse and granular immunoglobulin M (IgM) deposition on immunofluorescence microscopy [4]. Controversies continue regarding the classification of IgMN in the literature. Some previous articles reported that IgMN was associated with certain systemic disorders such as systemic lupus erythematosus, rheumatoid arthritis, and diabetes mellitus [5]. The disease had several similarities to both minimal change disease and focal segmental glomerulosclerosis, though some authors argued that there was no resemblance between IgMN and minimal change disease [6,7]. No standardized treatment has been suggested for IgMN so far. Agents reported in cases were steroids, a calcineurin inhibitor, and rituximab [3,8]. We aimed to present a young pregnant woman developing IgMN during pregnancy. 

## Case presentation

A 30-year-old woman was presented to the obstetric clinic of the hospital because of a complaint of edema in the lower extremities at the 31st week of pregnancy. Her earlier medical assessments revealed no abnormalities. This was her second pregnancy, having one abortus previously. There has been no record of renal illness in the family, having no other diseases, including diabetes mellitus, hypertension, or cardiac disorders. She was not using any routine medications, over-the-counter or herbal medicines. Tobacco, alcohol, or intravenous drug usage did not exist in history. The patient was consulted to the nephrology clinic because of the hematuria and proteinuria while being followed by the obstetric clinic. In the meantime, she underwent a cesarean section at 35 weeks of gestation because of beginning labor with breech presentation and fetal distress, delivering a baby with a birth weight of 1800 g. The patient was transferred to the nephrology clinic for planning renal biopsy on the third day after delivery.

In physical examination, her blood pressure was 110/70 mmHg. Bilateral +3 pitting edema existed in the pretibial region. Pulmonary, cardiac, and neurological assessments did not show any pathologic findings. She had no glomerular filtration rate (GFR) reduction, and her urine protein amount was 1852 mg/day. Hypoalbuminemia and hyperuricemia were prominent in lab analysis, in which lupus anticoagulant test and complement values were assessed within the normal range. There was no anemia or thrombocytopenia in the whole blood tests. Urinalysis showed +2 proteinuria and +3 hematuria with 21 red blood cells per high-power field. Autoimmune antibody tests were negative, except for anti-nuclear antibodies positive with >1/100 dilution. Viral serological assays came back with negative results. Table 1 shows the laboratory results on admission. In an ultrasonographic examination, an echogenicity compatible with two adjacent stones with a diameter of 5 mm was observed in the gallbladder. Free fluid existed around the gallbladder, reaching 43 mm in the Morrison space and 40 mm in the perisplenic area. Bilateral kidneys were of standard size, echo, and parenchymal thickness, with no stones or ectasia. Free fluid was determined in the abdomen at a depth of 50 mm between the intestinal loops. Pleural fluid was observed in the right and left hemithorax, reaching 65 and 44 mm in depth, respectively, with atelectatic lung segments next to the pleural fluid.

**Table 1:** Laboratory parameters of the patient eGFR, estimated glomerular filtration rate; CKD-EPI, chronic kidney disease epidemiology collaboration; IFAT, indirect fluorescent antibody test; HDL, high-density lipoprotein; LDL, low-density lipoprotein.

Parameter	Value	Reference	Parameter	Value	Reference
Glucose, mg/dL	101	70 - 110	Urine protein, mg/day	1852.2	< 300
Blood urea nitrogen, mg/dL	27	6 - 20	Triglyceride, mg/dL	175	0 - 200
Creatinine, mg/dL	0.8	0.5 - 0.9	Cholesterol, mg/dL	186	3 - 200
eGFR (CKD-EPI), ml/min/1.73 m^2^	99	> 60	HDL cholesterol, mg/dL	40	45 - 65
Aspartate aminotransferase, U/L	31	0 - 32	LDL-cholesterol (direct), mg/dL	122	0 - 130
Alanine aminotransferase, U/L	20	0 - 33	Parathormone, µg/L	46	15 - 65
Alkaline phosphatase, U/L	154	35 - 105	Folate, µg/L	20	3.89 - 26.8
Total Protein, g/L	46.4	64 - 83	Ferritin, µg/L	51	13 - 150
Albumin, g/L	26	35 - 52	Vitamin B12, ng/L	527	197 - 771
Total bilirubin, mg/dL	0.4	0.1 - 1.2	Complement C3, g/L	1.21	0.9 - 1.8
Direct bilirubin, mg/dL	0.2	0 - 0.3	Complement C4, g/L	0.18	0.1 - 0.4
Uric acid, mg/dL	10.8	2.6 - 6	Sedimentation, mm/ h	6	0 - 20
Calcium, mg/dL	8.9	8.6 - 10.2	Lupus anticoagulant screen ratio	0.89	< 1.2
Sodium, mmol/L	137	136 - 145	Lupus anticoagulant confirm ratio	0.86	
Potassium, mmol/L	5.8	3.5 - 5.1	Lupus anticoagulant normalized ratio	1.03	< 1.2
Chloride, mmol/L	106	98 - 107	Anti-nuclear antibody IgG (IFAT), >1/100 titer	Positive	
Phosphorus, mg/dL	4.8	2.45 - 4.5	White blood cell count, 10^3^/µL	7.66	4.5 - 10
Magnesium, mg/dL	2.08	1.6 - 2.6	Red blood cell count, x10^6^/µl	4.59	3.8 - 5.3
Gamma glutamyl transferase, U/L	10	6 - 42	Hemoglobin, g/dL	13.9	12 - 16
Lactate dehydrogenase, U/L	257	135 - 214	Hematocrit, %	42.7	36 - 44
Lipase, U/L	34	13 - 60	Platelet, 10^3^/µL	196	150 - 450

**Figure 1:**
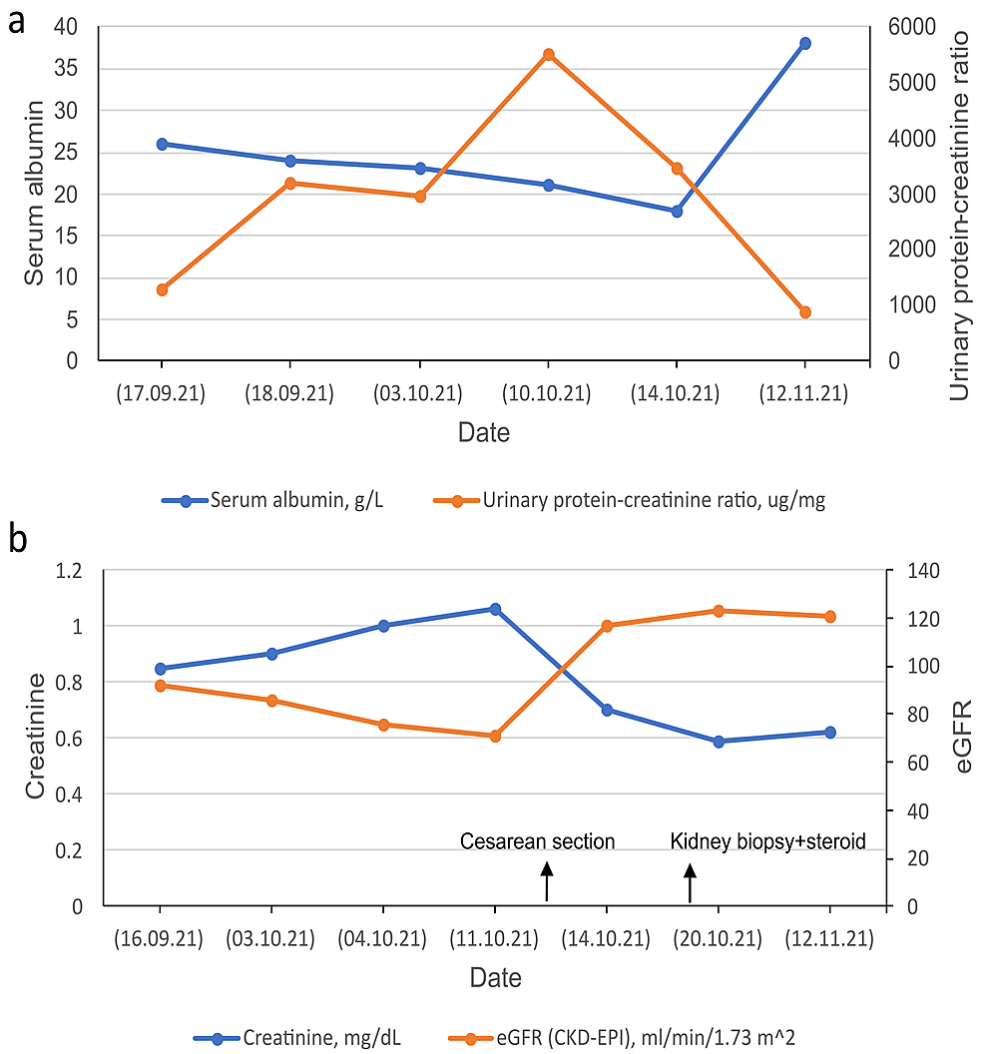
Serum albumin/proteinuria (a) and serum creatinine/eGFR (b) trajectories throughout the hospitalization period eGFR: estimated glomerular filtration rate, CKD-EPI: chronic kidney disease epidemiology collaboration equation

A renal biopsy was performed in the nephrology inpatient clinic after taking over from postpartum service on the 33rd day of admission to the hospital. Biopsy showed that 90% of tissue consists of cortex with a maximum of 45 glomeruli observed in serial sections. There was one sclerotic glomerulus, and the others had an increase in mesangial cells and membrane thickening, with a normal histological appearance of tube lumens. There was mild mononuclear inflammatory cell infiltration in the interstitial area. Vascular walls appeared normal. Histochemical studies revealed negative staining with Congo-red, periodic acid-Schiff, and Masson's trichrome. There were six glomeruli staining diffuse +3 with IgM in immunofluorescent studies, while other immune markers such as IgA, IgG, C1q, and C3 did not show staining in the rest of the glomeruli. Accordingly, the pathological examination was reported as IgMN with mesangial proliferation. 1 mg/kg/day prednisone therapy was started with 160-200 mg/day furosemide treatment, and she lost a total of 13 kg weight within a week. As her edema decreased without having any other complaints, she was discharged, suggesting an outpatient clinic control. Her eGFR (estimated glomerular filtration rate) was within the normal range in discharge. Figure 1a and Figure 1b show her serum albumin/proteinuria and creatinine/eGFR trajectories, respectively, throughout the hospitalization period.

## Discussion

We report a case of a pregnant woman displaying nephrotic syndrome in whom IgM nephropathy was identified. Pathologic assessment proved the mesangial IgM deposition in the kidneys, and steroid use led to remission after delivery with cesarean section. It should be considered that this case study has a drawback because of developing an uncommon lesion in a single patient. Moreover, we do not know whether pregnancy is a triggering factor for the disease. Thus, there is lacking evidence of whether the remission is due to the steroid or the delivery itself. The patient, therefore, should be monitored for any future development of other systemic diseases. A recent study of 57 patients with IgMN by Connor et al. reported that the median age was 40 years at presentation, older than that stated in prior studies signifying the highest frequency in childhood or adolescence. They also suggest that different gender and racial groups have a similar disease prevalence [2]. On the contrary, according to a study of 110 patients from Finland, IgMN is a disease of children and young adults, in which the mean age was 29 years, and 33% of patients were younger than 16 years of age [9]. Our patient was 30 years of age, approximately in line with these reported results.

There is insufficient knowledge regarding the etiology of the IgMN or what mechanisms underlie this disease. Lin et al. reported a significant correlation between in vitro IgM production by co-culture technique and IL-2 production in patients with IgMN, suggesting that the suppressor T-cell hyperfunction may prevent the shift of IgM B-cells to IgG B-cells, thereby increasing IgM in the patients [10]. Cavallo et al. suggested that patients with IgMN have immune reactants, which may act for an antigen-antibody interaction. Because of the permeability defect in IgMN, mesangial materials may prevent the clearing of macromolecular collections, leading to mesangial dysfunction. Mesangial deposits could fix the complement, thereby releasing the factors that intensify vascular permeability [11]. A study determined circulating heavy IgM in patients with IgMN, having a capacity of complement fixation activity [12]. Also, it has been postulated that the synthesis of autoimmune IgM antibodies participated in this process can be prevented effectively using rituximab therapy [13].

Nephrotic syndrome is a primary presentation of the disease in most patients. Hematuria or non-nephrotic proteinuria also is not uncommon, but most nephrologists do not prefer biopsy in these situations; lower patients, therefore, seem to have hematuria or proteinuria without nephrotic range [5,9]. Hence, the referral criteria and biopsy indication used by a nephrology clinic should be affecting the clinical presentation in retrospective series. A minority of patients in the recent series presented with nephrotic range proteinuria, and there were no presentations with isolated hematuria, contrary to previous reports [2,14]. Park et al. described a case of crescentic glomerulonephritis similar to mesangial IgA deposits in crescentic glomerulonephritis associated with IgA nephropathy as evidence that the disease can manifest in many different ways [3]. Furthermore, there were case reports concerning IgMN associated several diseases, including systemic lupus erythematosus [15], autoimmune hemolytic anemia [16], Wilson’s Disease [17], and Fabry disease [18]. Our patient presented for the first time with simple proteinuria and hematuria; nephrotic proteinuria developed subsequently during the hospital stay.

IgMN diagnosis is performed primarily by examining the renal biopsy sections using light microscopy, immunofluorescence staining, and electron microscopic analysis. Although there are no standardized diagnostic criteria worldwide, some criteria were used for IgMN definition, including mesangial IgM staining more than trace with or without IgA and IgG staining, and obvious mesangial deposits on electron microscopy. IgA and IgG staining, if any, should be lower intensity than IgM. Also, no systemic diseases should accompany these pathologic findings, such as systemic lupus erythematosus, rheumatoid arthritis, diabetes mellitus, paraproteinemia [2]. In a case series of 28 children, the main immunoglobulin accumulated was IgM in a diffuse pattern with the intensity ranging from +2 to +4. 78.6% of biopsies have IgM alone, 10.7% associated with C3, and 10.7% of biopsies associated with C3+C1q, without other immune deposits [19]. Mubarak et al. reported a case study of 57 adult patients in which glomerular mesangial cell proliferation was the most common morphologic change, with a diffuse mesangial positivity of IgM in all specimens. Subdominant IgA, C3, and C1q were found in 14.6%, 68.3%, and 51.2% of patients, respectively [20]. Our patient had extensive mesangial proliferation and +3 diffuse IgM staining without IgA, IgG, C3, and C1q staining.

The primary choice was the corticosteroids for the treatment of the disease for many years, while various agents have been used in inducing remission. Steroid response rate is reported between 39% and 80% in case series [2,9]. Myllymäki et al. used cyclophosphamide in steroid-resistant 22 patients, and 11 patients had a complete remission lasting at least four months [9]. Recently, rituximab has been used with successful results for second-line therapy in the event of steroid dependence or resistance. Downie et al. have administered 1 g rituximab two separate times without adverse reaction for a patient with frequent relapses, inducing remission successfully with cessation of prednisone [8]. Our patient exhibited complete remission using 1 mg/kg/day prednisone for now, and she was started to follow in the outpatient clinic.

## Conclusions

We described a case of IgMN in pregnancy, which entered remission with glucocorticoid therapy after delivery. The clinicopathological range of IgM nephropathy might be better comprehended through this report. More information could be gained from studies involving a larger sample size with longitudinal methods for a detailed explanation of IgMN. Our expectation in reporting this case was that clinicians should consider a potential IgMN diagnosis during pregnancy.
